# G-Protein Coupled Receptors Targeted by Analgesic Venom Peptides

**DOI:** 10.3390/toxins9110372

**Published:** 2017-11-16

**Authors:** James T. Daniel, Richard J. Clark

**Affiliations:** School of Biomedical Sciences, University of Queensland, Brisbane, QLD 4072, Australia; james.daniel@uqconnect.edu.au

**Keywords:** pain, GPCR, venom peptide, analgesia, *Conus*

## Abstract

Chronic pain is a complex and debilitating condition associated with a large personal and socioeconomic burden. Current pharmacological approaches to treating chronic pain such as opioids, antidepressants and anticonvulsants exhibit limited efficacy in many patients and are associated with dose-limiting side effects that hinder their clinical use. Therefore, improved strategies for the pharmacological treatment of pathological pain are urgently needed. G-protein coupled receptors (GPCRs) are ubiquitously expressed on the surface of cells and act to transduce extracellular signals and regulate physiological processes. In the context of pain, numerous and diverse families of GPCRs expressed in pain pathways regulate most aspects of physiological and pathological pain and are thus implicated as potential targets for therapy of chronic pain. In the search for novel compounds that produce analgesia via GPCR modulation, animal venoms offer an enormous and virtually untapped source of potent and selective peptide molecules. While many venom peptides target voltage-gated and ligand-gated ion channels to inhibit neuronal excitability and blunt synaptic transmission of pain signals, only a small proportion are known to interact with GPCRs. Of these, only a few have shown analgesic potential in vivo. Here we review the current state of knowledge regarding venom peptides that target GPCRs to produce analgesia, and their development as therapeutic compounds.

## 1. Introduction

Animal venoms constitute one of the largest and most diverse natural libraries of bioactive peptide molecules, many of which have been evolutionarily optimised to target physiologically important proteins (ion channels, receptors and transporters) with high affinity and selectivity [1,2]. Since many of these membrane proteins are closely conserved from invertebrates to higher mammals, venoms offer a rich reservoir for discovery of novel compounds with potential applications as molecular probes for biomedical research and lead molecules for development of pharmaceuticals [1,3]. Peptide toxins often possess several desirable pharmaceutical qualities such as ease of chemical synthesis and modifications, and small, stable three-dimensional structures. A particularly appealing application of peptide toxins as drugs is for the treatment of chronic pain, which is currently poorly managed clinically. Many venom constituents have evolved to target ion channels and receptors that mediate fast synaptic transmission (e.g., voltage-gated sodium and calcium channels) to elicit rapid immobilisation of their prey or predators. Such proteins are also key players in neurotransmission of pain signals and a plethora of venom peptides are currently in development as analgesics which target ligand- or voltage-gated ion channels to rapidly reduce neuronal excitability associated with pain. In contrast, G-protein coupled receptors (GPCRs) are relatively unexploited as analgesic targets for therapeutic peptide development, despite being the largest family of membrane-bound receptors, the most commonly targeted class of receptors by current pharmaceuticals and crucial regulators of both canonical and pathological pain. Nevertheless, several venom peptides have been discovered that target GPCRs and are in development as analgesic compounds to treat symptoms of chronic pain. In this article, we outline the physiological and pathological role GPCRs play in pain signalling and review the mechanisms by which known GPCR-targeting venom peptides achieve analgesia and their progress as clinical compounds. 

## 2. GPCR Modulation of Nociception 

### 2.1. GPCR Signalling Pathways

GPCRs are characterised by seven transmembrane (TM) domains and are the largest family of cell surface proteins involved in most if not all physiological processes by transducing extracellular signals into a cellular response. GPCRs are coupled to heterotrimeric guanosine triphosphate-binding protein subunits (G proteins) and following activation, undergo conformational changes resulting in G-protein dissociation. The liberated G-protein subunits (α monomer and βγ dimer) subsequently activate several distinct signalling cascades depending on the G-protein subtypes. In neurons, the primary Gα protein subtypes that stimulate downstream effects of GPCR activation are Gα_q/11_, Gα_s_ and Gα_i/o_. 

The Gα_q/11_ subunit is primarily coupled to excitatory GPCRs and activates phospholipase C (PLC), particularly the PLCβ isoform, which cleaves phosphatidylinositol 4,5-bisphosphate (PIP2) into inositol triphosphate (IP3) and diacylglycerol (DAG). IP3 and DAG subsequently activate protein kinase C (PKC) and cause a transient increase of intracellular Ca^2+^. 

The other main excitatory subtype, Gα_s_, is coupled to adenylate cyclase (AC), an enzyme which generates cyclic adenosine monophosphate (cAMP) from adenosine triphosphate (ATP). Increased concentration of cAMP acts as a second messenger resulting in activation of many cAMP-dependent kinases, particularly protein kinase A (PKA). These and other kinases mediate diverse and complex cellular functions by phosphorylating ion channels and transcription factors, typically mitogen-activated protein kinase (MAPK) and extracellular signal-regulated kinase (ERK). 

The Gα_i/o_ subtype mediates the opposite effect to Gα_s_ through inhibition of AC activity and cAMP accumulation, also acting to modulate various effector cascades. Importantly for pain, the Gβγ subunit of many Gα_i/o_-linked GPCRs, such as opioid receptors (ORs) and γ-aminobutyric acid receptors (GABA_B_Rs), are known to directly modulate the activity of ion channels. Specifically, inhibition of voltage-gated calcium channels (Ca_V_s) and augmentation of G-protein coupled inwardly rectifying potassium channel (GIRK) activity in central and peripheral neurons are thought to be key mechanisms underlying GPCR-mediated analgesia [4,5]. GPCRs can also signal via non-G-protein-mediated pathways including auxiliary proteins and internalisation apparatus such as β-arrestin. The complexity of GPCR signalling is further expanded by emerging concepts such as allosteric modulation and functional selectivity (or signalling bias) in which the same receptor triggers different molecular cascades in response to different ligands. These signalling pathways underlie many aspects of normal and pathological pain detection and processing which are outlined below using key examples. 

### 2.2. Role of GPCRs in Acute Pain 

#### 2.2.1. Peripheral Nociception

Detecting noxious environmental stimuli is a key mechanism for avoiding or limiting physical damage and is paramount to the survival of most organisms. In animals, external pain signals are detected and transmitted by a subset of peripheral somatosensory neurons known as nociceptors [6]. These nerve fibres, which originate in the dorsal root ganglion (DRG), project throughout the body to innervate skin, visceral tissue and muscle and are broadly divided into the myelinated “fast” Aδ fibres and unmyelinated “slow” C-fibres [7]. Nociceptors are enriched with a vast array of specialised ion channels and receptors that respond differentially to noxious thermal, mechanical and chemical stimuli enabling transmission of external pain signals to the central nervous system (CNS) [6,8]. The initiation of a pain signal in peripheral nociceptors is carried out primarily by ionotropic transducer proteins such as the archetypal transient-receptor potential (TRP) family, acid-sensing ion channels (ASICs), P2X and K_2_P channels, which are activated by noxious stimuli and allow cation influx to generate membrane potentials. The signal is propagated along the cell body toward the DRG by voltage-gated sodium (Na_V_) and potassium channel (K_V_) subtypes, many of which are selectively expressed on nociceptive fibres (e.g., Na_V_1.8, Na_V_1.9). Activation of presynaptic Ca_V_s generates inward Ca^2+^ currents and subsequently activates Ca^2+^-dependent kinases leading to neurotransmitter exocytosis. Although voltage- and ligand-gated ion channels are generally reputed as the major membrane proteins involved in nociceptive neurotransmission, nociceptors also heterogeneously express numerous families of GPCRs, both pre- and post-synaptically, which heavily modulate this process [9,10,11]. Notable GPCR families expressed by nociceptors include metabotropic glutamate receptors (mGluRs), ORs, GABA_B_Rs, muscarinic acetylcholine receptors (mAChRs), α-adrenergic receptors (α-ARs), serotonin (5-HT) receptors and a variety of chemokine receptors (CXC/CCR) [7,9]. These and other GPCRs act to transduce extracellular signals from diverse endogenous and exogenous ligands (e.g., neurotransmitters, inflammatory mediators, pharmaceutical compounds) which alter excitability and expression of key ionotropic proteins, controlling short- and long-term changes in synaptic activity of nociceptors. 

#### 2.2.2. Central Processing

DRG neurons communicate peripheral pain information centrally through synaptic inputs into laminae I-IV of the dorsal horn. At these locations, nociceptive afferents release several types of excitatory neurotransmitters onto central “second-order” neurons, predominantly glutamate, which acts at ionotropic α-amino-3-hydroxy-5-methyl-4-isoxazolepropionic acid (AMPA) and *N*-methyl-d-aspartic acid (NMDA) receptors, and metabotropic glutamate receptors (mGluR), and the neuropeptides substance P and calcitonin gene-related peptide (CGRP), which act via the GPCRs neurokinin 1 and calcitonin receptor-like receptor (CRLR), respectively [7,12]. Subsequent generation of excitatory post-synaptic potentials (EPSCs) in second-order neurons carries pain information along supraspinal and spinothalamic tracts which project onto the brainstem and thalamic nuclei and ultimately communicate with cortical and subcortical regions to produce an integrated physical and emotional response [12,13]. Descending pathways from these regions project back to second-order neurons and provide inhibitory control primarily through GABAergic, serotonergic, adrenergic and glycinergic feedback circuits [14]. Most major endogenous neurotransmitters in both central ascending (e.g., glutamate, acetylcholine) and descending (e.g., 5-HT, GABA, glycine) pain pathways elicit fast synaptic transmission over millisecond time scales by activating post-synaptic ligand-gated ion channels (e.g., AMPA, nAChR, 5-HT_3_, GABA_A_R) but often also act at one or more GPCR targets (e.g., mGluR, 5-HT_1_, GABA_B_R, mAChR), which mediate more gradual changes in membrane excitability and activation kinetics over seconds to minutes via intracellular signalling cascades. The complex interplay between different GPCR pathways is a delicate balance and as such, dysfunctional GPCR activity in pain circuitry can have severe physiological consequences.

### 2.3. Role of GPCRs in Pathological Pain 

Despite the importance of acute pain for survival, pain can also be chronic and may persist for long periods (months to years) even in the absence of stimuli. Various triggering events including diseases (e.g., cancer, diabetes mellitus, inflammatory bowel syndrome), exogenous chemicals (e.g., cytotoxic and neurotoxic medications) and traumatic physical injuries can lead to the development of persistent chronic pain symptoms. In many cases the source of chronic pain can be traced back to pathological alterations in the structural and molecular properties of nociceptive neurons, termed “neuropathic” pain. However, the manifestation of neuropathies is highly dependent on the location and severity of initial pathogenic events and individual risk factors such as age, gender and genetic background, leading to complex and heterogeneous patient phenotypes which complicates the clinical management of chronic pain [15,16]. The most common symptoms include moderate to severe mechanical and thermal hyperalgesia (increased sensitivity to pain), allodynia (painful response to innocuous stimuli), spontaneous pain and persistent pain, which may be localised or widespread. Epidemiological estimates indicate up to 20% of the population may suffer from moderate to severe chronic pain symptoms with 7–10% displaying neuropathic characteristics [16,17,18,19,20]. Accordingly, chronic pain conditions carry a considerable economic burden with estimated costs between $560 and $635 billion in the United States annually [21].

The molecular mechanisms underlying development of chronic neuropathic pain are highly complex and the subject of ongoing research. It has been well established that the increased local concentration of aberrant chemicals associated with injury or disease, particularly inflammatory mediators, can induce profound and long-term alterations of the expression and function of nociceptive proteins, leading to sustained pathological hyperexcitability of peripheral nociceptors; a process known as sensitisation [6,7,8]. Since GPCRs are key regulators of canonical nociceptor function, it is not surprising that they are also heavily involved in sensitisation and pain pathogenesis. Many sensitising biochemicals in the inflammatory milieu act through GPCRs, primarily those coupled to excitatory Gα_q_ and Gα_s_ signalling pathways, to induce increased nociceptor excitability and lower thresholds for activation. For example, activation of bradykinin receptors (B1 and B2) [22,23], prostaglandin E2 (PGE_2_) receptors (EP_1_ and EP_4_) [24], histamine receptors (H_1_ and H_2_) [25], purinergic receptors (P2Y) [26], protease-activated receptor 2 (PAR2) [27], and various chemokine receptors [28] by their respective ligands have been shown to influence activation kinetics of nociceptors and contribute to long-term sensitisation associated with neuropathic pain. Intracellular signalling pathways initiated by these receptors converge on key downstream effector proteins which alter membrane targeting and gene expression of surface proteins in peripheral nociceptors. Protein kinase C (PKC) isoforms play an important role in shifting activation thresholds of nociceptors to more depolarised potentials, contributing to development of neuropathic pain [29,30]. PKA, MAPK and ERK have also been implicated in increasing nociceptor excitability since genetic knockout or pharmacological inhibition of these kinases hinders development of hyperalgesia and allodynia in response to sensitising agents [24,31,32,33]. Important nociceptive ion channels, principally TRP, Ca_V_ and Na_V_ channels, are a further point of convergence for GPCR-mediated pathways. Accordingly, increased PKC activity has been shown to upregulate the sensitivity and exocytosis of TRPV1 [34,35], Ca_V_ α_2_δ subunits [36] and tetrodotoxin-resistant (TTX-R) Na_V_ activity [37] in sensory neurons. 

GPCRs also facilitate sensitisation by promoting maladaptive neuroplastic alterations in central pain pathways. Repeated pathological firing of nociceptive afferents onto spinal neurons triggers long-term potentiation (LTP) and reinforced transmission of pain signals centrally, which in part is facilitated by G-protein signalling [7]. Illustrative of this process, activation of mGluR_1_ and mGluR_5_ has been shown to strengthen synaptic connections in dorsal horn neurons by ERK-mediated phosphorylation of ion channels, and contribute to development of pain symptoms [38,39,40]. Loss of descending monoaminergic inhibitory control through dysfunction of adrenoceptors and 5-HT receptors is another GPCR-dependent mechanism by which pathological increases in pain transmission may arise in central sensory nerve tracts [14,41]. Ultimately, the combination of peripheral and central sensitisation events facilitated by pathological GPCR activity can lead to a radically altered molecular landscape in neural pain circuits and contributes heavily to the development of chronic pain symptoms in vivo. The significant cellular and molecular heterogeneity observed in both healthy and pathological nociceptive pathways and complex aetiology of neuropathic pain presents a major obstacle for clinical management. Therefore, identifying common mechanisms among distinct neuropathies which can be exploited to ameliorate pathological nociceptor hyperexcitability is a major goal of current therapeutic strategies to treat chronic neuropathic pain. 

### 2.4. GPCRs as Analgesic Targets

GPCRs comprise the largest and most diverse gene superfamily of membrane receptors targeted by current pharmaceuticals to modulate disease progression or symptoms [42], and given the ubiquitous role GPCRs play in modulating physiological and pathological neurotransmission in the mammalian CNS, targeting these receptors has obvious merit for the treatment of neuropathic pain. The three major classes of first-line pharmaceutical compounds used for clinical management of neuropathic pain are opioids, antidepressants and anticonvulsants, which predominantly act through GPCRs, transporters and ion channels, respectively [15]. Although direct inhibition of ion channel activity is an appealing strategy to produce rapid reduction of membrane excitability, indirect modulation of nociceptor activity by GPCRs, especially those coupled to Gα_i/o_ subunits owing to their inhibitory downstream effects, can be exploited for the reduction of pathological nociceptor hyperexcitability and alleviation of pain signalling. Indeed, most putative GPCR targets that exhibit analgesic properties upon activation are coupled to Gα_i/o_-mediated pathways [9,10]. 

The therapeutic utility of GPCRs in pain treatment is excellently illustrated by the opioid receptors (ORs), which are among the most clinically successful and well-characterised analgesic targets. For years, opioids derived from the opium poppy (e.g., morphine and codeine) and their synthetic derivatives (e.g., fentanyl and tramadol), which act as agonists at ORs, have been considered the gold standard in pain relief and remain the preeminent and most widely used class of analgesic compounds for treatment of acute and chronic pain [43]. Three major OR subtypes (μ, δ and κ), which all couple to Gα_i/o_, are expressed throughout the mammalian nervous system and digestive tract where they are activated by endogenous opioid peptides and regulate a variety of neurophysiological processes including pain sensation, reward, cognition, digestion and respiration [44,45]. The overall inhibitory physiological effects of opioid administration, particularly analgesia, involves Gβγ-mediated inhibition of Ca_V_ activity and activation of GIRKs, which ultimately results in reduced neuronal excitability throughout both the central and peripheral nervous systems [5,45,46]. However, although ORs are well-established analgesic targets, use of opioids to treat pain comes with several limitations. Prolonged opioid administration causes ORs to undergo extensive desensitisation, resulting in increased tolerance over time, and larger doses required to overcome this effect are associated with increased severity of dangerous side effects such as respiratory depression [47]. Moreover, long-term opioid use can lead to powerful psychological and physiological withdrawal symptoms which contribute to development of opioid dependence, and are associated with increased risk of prescription opioid-related drug abuse and hospitalisations, placing further burden on the health system [48,49]. 

Ca_V_s are a particularly well-documented analgesic target in neurons, since their activation is essential for Ca^2+^ influx and neurotransmitter release, and they are a key effector of most known Gα_i/o_-coupled GPCRs. Accordingly, many analgesic compounds approved for clinical use act to directly inhibit Ca_V_ activity, such as the anticonvulsants gabapentin and pregabalin, as well as the only venom peptide approved for treatment of neuropathic pain, ziconotide [50,51,52,53]. However, like those outlined above, these pharmaceuticals also exhibit narrow therapeutic windows due to substantial dose-limiting side effects from inhibition of central synaptic transmission, as well as lack of efficacy in many patients [54]. Therefore, specifically targeting the peripheral component of pain to reduce central side effects is a major goal for the development of effective and selective pain therapeutics. Interestingly, there exists a splice variant of Ca_V_2.2 known as e37a which is selectively expressed on nociceptive neurons and is susceptible to G-protein-mediated inhibition [55,56]. In this sense, selectively targeting Ca_V_s in pain pathways via GPCRs may have advantages over direct Ca_V_ inhibition, such as improved side-effect profiles and therapeutic windows. 

Another approach to treating chronic pain is to strengthen descending inhibitory control of nociceptive pathways by increasing local concentrations of key GPCR-targeting monoaminergic neurotransmitters involved in this process, namely serotonin and norepinephrine acting via their inhibitory receptor subtypes such as 5-HT_1_ and α_2_Rs. Incidentally, antidepressant pharmaceuticals including tricyclic antidepressants (TCAs), serotonin–norepinephrine reuptake inhibitors (SNRIs) and selective serotonin reuptake inhibitors (SSRIs) act to block uptake and increase synaptic accumulation of either or both serotonin and norepinephrine in descending pathways and blunt central pain signals [57]. Accordingly, antidepressants are a major class of drugs used in the pharmacological management of chronic pain, but only show limited efficacy in many patients and are also hindered by neurological side effects [14,57].

Overall, the current outcomes of the various pharmacotherapies for neuropathic pain patients are generally poor. In addition to their numerous dose-limiting side effects, first-line analgesics are only effective in about 40–60% of neuropathic pain patients and a large proportion become refractory to these treatments over time [17,58]. The apparent unmet demand for efficacious analgesic compounds highlights the necessity for improved or novel pharmacological strategies to treat neuropathic pain conditions. Owing to the complex and multifaceted functions of GPCRs, there is significant room for novel or improved ligands that modulate GPCR targets. Conversely, these same properties can also make isolating specific pathways related to analgesia, without perturbing other canonical functions, problematic. Nevertheless, continuous technological improvements in methods used to characterise structural and mechanistic properties of GPCRs, such as the unprecedented availability of high-resolution GPCR crystal structures, has provided deeper insights into the nuances of GPCR signalling (e.g., allosteric modulation, functional selectivity, desensitisation and internalisation), which further expands the possible avenues for development of novel analgesic strategies. Thus, despite the clinical shortcomings of conventional GPCR ligands like opioids, GPCR-mediated analgesia remains an appealing avenue for pharmacological therapy of chronic neuropathic pain. 

## 3. Analgesic Venom Peptides Targeting GPCRs

### 3.1. Development of Venom Peptides as Analgesic Drugs

In light of the failings of current small-molecule analgesics and significant societal impacts of poorly managed pain, improved pharmacological compounds which selectively modulate pathological pain signalling are needed. A major traditional and contemporary strategy for novel drug discovery is to screen naturally occurring compounds and select those with novel or desirable pharmacodynamic properties. In this regard, the remarkable chemical and pharmacological diversity of animal venoms presents an ideal starting point. In contrast to synthetic combinatorial approaches, proteinaceous venom constituents offer the advantage of having been ‘pre-optimised’ by nature following millions of years of evolutionary selection for toxins that potently and specifically target important physiological pathways. Peptide toxins isolated from venomous taxa which use rapid immobilisation as a primary trophic strategy, such as spiders, scorpions and cone snails, are especially good candidates for analgesic development because their venoms have been selected for inhibition of fast synaptic transmission [2]. Accordingly, venom peptides from these and similar species have been the subject of intense investigation over recent decades, and parallel advances in technologies used for discovery and molecular characterisation have enabled unprecedented access to the diverse biochemical universe of venom peptides and greatly expanded the number venom peptide-based drug leads in preclinical and clinical development [1,3,59,60]. As highlighted in Table 1, several venom peptides are now approved as therapeutics for diverse clinical indications including cardiovascular dysfunction (hypertension and angina), neuropathic pain and type 2 diabetes mellitus. 

The traditional discovery pipeline involves isolation of peptides from venom extracts by chromatographic fractionation (typically reverse-phase high-performance liquid chromatography (RP–HPLC)) and proteomic characterisation using mass spectrometry (MS)-based approaches. Isolation of peptide constituents is combined with pharmacological screening using particular bioassays including in-vitro high-throughput cell-based assays to determine toxin–protein interactions, ex-vivo tissue preparations (e.g., sensory nerves) and/or in-vivo animal models of disease to determine their physiological and potential therapeutic effects [61]. The sequence of “hit” peptides (i.e., those with favourable pharmacological properties) is then derived using proteomic methods such as MS/MS or Edman degradation. However, such strategies are often laborious and cost-intensive and thus recent advances in next-generation sequencing techniques have heralded a shift towards molecular biology approaches for venom peptide discovery. The advantage of genomic techniques is the ability to generate cDNA libraries from venom glands cheaply and efficiently, which can be mined in silico to determine novel peptide sequences directly from their encoding genes, offsetting the requirement for direct access to crude venom samples. However, a major limitation is the inability to predict disulfide arrangements and other post-translational modifications. Therefore, a combination of proteomic and genomic approaches, collectively termed “venomics”, has proved most effective for interrogating venom diversity and isolating lead molecules for preclinical development [60,62].

Other than their high target specificity, venom peptide molecules often possess several other desirable pharmaceutical properties. Their generally short chain lengths (<50 amino acids) make them highly amenable to chemical synthesis and thus putative bioactive peptide sequences identified by proteomic and/or genomic approaches can be readily synthesised in large quantities using solid-phase peptide synthesis (SPPS) [63,64]. This is particularly important for subsequent structural and preclinical studies which require a greater quantity of peptide than is usually accessible from crude venoms. Moreover, it allows incorporation of synthetic modifications to manipulate the biochemical properties of venom peptides and aid their development as pharmaceuticals. Another important characteristic of many venom peptides is their compact and rigid three-dimensional structures. Since most venoms are delivered parenterally through various types of envenomation apparatus (e.g., fangs/teeth, harpoon, barbs), the bioactive constituents must retain structural integrity and resist degradation in harsh endogenous environments long enough to elicit physiological effects. Nature has developed an elegant solution to this problem through incorporation of multiple cysteine residues and post-translational formation of disulfide bonds, which stabilise the topological conformation required for specific receptor interactions and also confer some resistance to proteolytic degradation in vivo [65]. Many disulfide arrangements are conserved between diverse taxa (e.g., the inhibitory cysteine knot (ICK) conformation [66]), suggesting that, analogous to combinatorial chemistry techniques, these privileged disulfide frameworks act as ancestral scaffolds enabling extensive diversification of non-cysteine residues and giving rise to classes of structurally similar but functionally distinct peptides [67]. Another advantage of the constrained structures adopted by peptide toxins is the ability to perform highly detailed structural characterisation by nuclear magnetic resonance (NMR) spectroscopy which, in conjunction with bioassays, is vital for the structure-activity-based development of drug leads from venoms [68]. In spite of their favourable drug-like properties, like many peptide-based drugs, the clinical application of venom peptides is often hindered by short metabolic half-lives, low membrane penetration, poor oral bioavailability and lack of efficacy in vivo [59,69]. Therefore, numerous synthetic strategies have been developed and applied to venom peptide leads, such as disulfide substitutions, cyclisation, truncations and conjugations, among others, with the aim of engineering peptide leads with improved pharmaceutical properties [70,71,72]. 

The diverse physiological functions of GPCRs suggest that their modulation by venom peptides might have a similarly diverse range of research and clinical applications. Indeed, a number of venom peptides that target a variety of GPCRs have been discovered in recent decades [73], including the clinically approved glucagon-like peptide 1 receptor (GLP-1R) agonist exenatide, a synthetic version of the Gila monster (*Heloderma suspectum*) venom peptide, exendin-4 [74,75]. In terms of analgesia, however, the majority of peptide toxins undergoing development as treatments for pain directly interact with ionotropic targets to modulate membrane excitability, which is not surprising given their crucial role in neurotransmission and conservation among diverse evolutionary lineages. Key examples include Na_V_ inhibitors, Ca_V_ inhibitors, K_V_ inhibitors, ASICs inhibitors and nAChR inhibitors [76]. In contrast, comparatively fewer venom toxins that display analgesic properties act via GPCRs. This disparity could be reflective of a bias in target-based peptide discovery which favours the already established ionotropic analgesic targets. Moreover, depending on the species and primary physiological effects envenomation, the venom itself may contain a far greater proportion and quantity of peptides directed towards ion channels (e.g., cone snails). Despite being overshadowed by peptides targeting ionotropic membrane proteins in terms of both research focus and venom composition, several GPCR-targeting venom peptides have shown promise as analgesic leads and are outlined below. 

### 3.2. Conopeptides Targeting GPCRs with In-Vivo Analgesic Efficacy 

Carnivorous marine gastropods of the *Conus* genus (cone snails) are renowned for their potent neurotoxic venoms, which are potentially lethal to humans. These slow-moving molluscs have evolved an intriguing prey capture and predator defence strategy whereby a harpoon-like radula tooth is propelled into nearby victims enabling parenteral delivery of highly paralytic venoms [77]. The bioactive constituents of cone snail venoms comprise a plethora of peptide toxins, termed conopeptides, which target an astounding array of membrane proteins involved in neurotransmission including voltage-gated (e.g., Ca_V_, Na_V_, K_V_) and ligand-gated ion channels (e.g., nAChRs, ASICs), GPCRs (e.g., GABA_B_R, α-AR) and transporters (e.g., NET) [64,78]. Proteomic analyses of *Conus* venoms have revealed over 1000 unique conopeptide sequences in some species [79]. With >700 species estimated to exist in the wild, *Conus* venoms are one of the richest known sources of bioactive peptide compounds, of which only 0.1% is estimated to be structurally and functionally characterised [64]. Those conopeptides that are rich in disulfide bonds (≥2) are referred to as conotoxins and are generally the most abundant within *Conus* venoms. Major pharmacological classes of conotoxins include ω-conotoxins (Ca_V_ inhibitors), α-conotoxins (nAChR antagonists and GABA_B_R agonists), µ-conotoxins (Na_V_ inhibitors), κ-conotoxins (K_V_ inhibitors) and χ-conotoxins (NET antagonists). A variety of disulfide-poor (1 or no disulfides) peptides are also present within *Conus* venoms and include the conantokins (*N*-methyl receptor antagonists), contulakins (neurotensin receptor agonists), conopressin (oxytocin receptor agonists and V_1A_ vasopressin receptor antagonists), conorphins (κ-opioid receptor agonists) and contryphans (ion channel inhibitors). The synergistic actions of these diverse inhibitory conopeptides result in reduction of neuronal excitability throughout the nervous system and rapid immobilisation, or even death, of predators or prey. Despite the neurotoxic effects of envenomation, by virtue of their specific interactions with neuronal membrane proteins, many individual conopeptides have been adopted as neurophysiological probes with applications as research tools and therapeutic leads. In this regard, the success of conopeptides is best illustrated by the 25-amino-acid ω-conotoxin, MVIIA (ziconotide), which is a potent and selective antagonist for the N-type Ca_V_ (Ca_V_2.2) and was approved in 2004 for clinical use in intractable neuropathic pain [54]. Interestingly, the only GPCR-targeting venom peptides which exhibit in-vivo analgesic efficacy to date have been isolated from species of *Conus*. 

#### 3.2.1. GABA_B_ Receptor Targeted by α-Conotoxins

The GABA_B_R is a heterodimeric GPCR which acts as the metabotropic receptor for inhibitory neurotransmitter GABA, and is the target of the skeletal muscle relaxant baclofen, used to treat spasticity [80,81,82,83]. These receptors exist as heterodimers of GBR1 and GBR2 subunits and belong to the Class C GPCR family, also including mGluRs, which are characterised by a large extracellular Venus Fly Trap domain (VFTD) that contains the ligand binding site [84]. Specifically, the GBR1 VFTD binds orthosteric agonists and antagonists [85,86] while the interaction of the GBR2 ectodomain acts to increase ligand affinity and stabilise cell surface expression [87,88]. The TM domain of GBR2 also links receptor activation to G-protein signalling [89]. Crystal structures of the individual GABA_B_R subunits and heterodimer have been solved and reveal a unique structural mechanism for activation in which agonist binding stabilises the closed conformation of the GBR1 subunit [90,91]. Native GABA_B_R heterodimers exist in complexes with auxiliary proteins belonging to the potassium channel tetramerisation domain (KCTD) family which regulates G-protein activation kinetics [92]. Subsequent activation of Gα_i/o_ acts to inhibit adenylate cyclase activity and cAMP accumulation and regulate kinase activity, while the dissociated Gβγ subunit modulates the function ion channels to elicit inhibition of membrane excitability. Like other Gα_i/o_-coupled GPCRs, inhibition of P/Q-type (Ca_V_2.1), Ca_V_2.2 and R-type (Ca_V_2.3) Ca_V_s is a key functional outcome of GABA_B_R activation and has been observed in many central and peripheral neuronal cell lines [93,94,95,96,97,98]. Another important ionotropic target of GABA_B_Rs are the post-synaptic GIRKs which are activated by the Gβγ subunit to produce membrane hyperpolarisation, contributing to inhibition of synaptic transmission [99,100,101]. 

GABA_B_Rs are expressed on both pre- and post-synaptic terminals of peripheral and central neurons with particularly robust expression in the thalamus, hippocampus and cerebellum [100], dorsal laminae of the spinal cord and peripheral DRG neurons [102]. The inhibitory actions of GABA_B_R activation at these sites mediate several neurological processes such as sedation, cognition, digestion, reward and pain, and this receptor has also been implicated in certain neurological disorders including epilepsy and addiction [83,103,104]. Accordingly, administration of baclofen has strong sedative, anti-nociceptive and muscle relaxant properties [103], which are inhibited by antagonists [105], while knockout of either the GBR1 or GBR2 subunit in mice causes spontaneous seizures, hyperalgesia, hyperlocomotion and impaired memory [106,107,108]. Baclofen is currently the only clinically approved GABA_B_R agonist, typically administered intrathecally to treat severe spasticity associated with diseases such as cerebral palsy and multiple sclerosis [83]. In terms of pain, GABA_B_R activation has been repeatedly identified as a mechanism for reversing hyperalgesia and allodynia in various models of acute [109,110] and chronic pain [111,112,113], through inhibitory pre-synaptic actions on peptidergic nociceptive afferents [114] and dorsal horn neurons [115,116]. However, tolerance to the anti-nociceptive effects of baclofen develops rapidly which, along with its substantial sedative side effects, precludes its clinical use for non-spastic pain treatment.

The α-conotoxins are a pharmacological family of conotoxins traditionally characterised by their antagonistic activity at nAChRs. Most α-conotoxins belong to the A-superfamily of *Conus* toxins and exhibit a type I cysteine framework (CC-C-C) in a native CysI–III, CysII–IV disulfide connectivity. Early α-conotoxins (e.g., GI and MI) were found to potently inhibit neuromuscular currents mediated by nAChR subtypes [117,118,119], however, many later-discovered α-conotoxins showed selectivity for neuronal nAChR subtypes such as α7-selective ImI [120], α3β4-selective AuIB [121], and α9α10-selective Vc1.1 and RgIA [122]. Neuronal nAChRs are ligand-gated ion channels comprising pentameric combinations of α_1_–α_10_, β_2_–β_4_, γ, δ or ε subunits that are distributed throughout the sensory nerve tracts, and their dysfunction has been implicated in a variety of neurological disorders including Parkinson’s disease, addiction, depression and pain [123,124]. Accordingly, α-conotoxins that target neuronal nAChRs have been thoroughly investigated for their potential therapeutic properties, particularly as analgesics [125,126]. Several α-conotoxins with different subtype selectivity profiles including Vc1.1, AuIB, RgIA and MII produce analgesic effects and contribute to functional recovery in various rodent models of neuropathic pain including chronic constriction injury (CCI), partial nerve ligation (PNL), chronic visceral hypersensitivity (CVH) and chemotherapy, without developing tolerance or cognitive side effects [102,122,127,128,129,130,131,132,133]. One particular α-conotoxin, Vc1.1, entered clinical trials (ACV1) for the treatment of sciatic neuropathic pain but was withdrawn at phase IIA based on observations of reduced in-vitro potency at human versus rat α9α10 receptor [134]. 

In addition to their inhibition of nAChRs, a subset of α-conotoxins which includes Vc1.1, RgIA, AuIB and PeIA, but not ImI or MII, was found to also inhibit Ca_V_-mediated currents in rodent DRG neurons [129,135,136]. This inhibition was not mediated by direct interaction with the Ca_V_2.2, since no effect was seen in *Xenopus* oocytes or HEK293 expressing Ca_V_2.2 alone [135,137]. Moreover, α-conotoxin inhibition of Ca_V_2.2 was abolished in the presence of a non-hydrolysable GDP analogue, pertussis toxin and a c-Src kinase inhibitor, suggesting that this effect is mediated by a Gα_i/o_-linked GPCR pathway [135]. Application of a suite of selective GPCR antagonists revealed that only GABA_B_R antagonists CGP 55845, CGP 54626 or phaclofen significantly reduced inhibition of Ca_V_2.2 by α-conotoxin Vc1.1, and this peptide did not produce an additive effect following application of selective GABA_B_R agonist baclofen, indicating that inhibition of Ca_V_2.2 is mediated by activation of GABA_B_R [135]. In further support of this paradigm, Cuny et al. demonstrated siRNA knockdown of either the GBR1 or GBR2 subunit in DRG neurons also significantly lowered the blockade of Ca_V_2.2 by Vc1.1, RgIA and AuIB [137]. Additionally, transfection of Ca_V_2.2 and GABA_B_R subunits in HEK293 was sufficient to reconstitute the inhibitory activity of Vc1.1. This mechanism appears to play a major role in α-conotoxin analgesia because administration of selective GABA_B_R antagonists CGP 55845 or SCH 50911 completely abolishes the analgesic effects of Vc1.1 in CCI, PNL and CVH models of pain [102,128,129]. The sequence and pharmacological properties of several key α-conotoxins are summarised in Table 2.

The GABA_B_R is an appealing therapeutic target and its activation is known to evoke pain-relieving effects, however, its use in pain treatment is limited by neurological side effects. Therefore, novel compounds which activate GABA_B_R-mediated signalling pathways with selectivity toward the analgesic component may offer new therapeutic leads for treating pain. Interestingly, in contrast to conventional agonists, α-conotoxins appear to exhibit functional selectivity at GABA_B_R and inhibit Ca_V_s through a novel mechanism. Unlike the Gβγ-mediated Ca_V_ inhibition in response to conventional agonists which occurs predominantly in a voltage-dependent manner and can be overcome by a strong depolarising pre-pulse [140], α-conotoxins elicit Ca_V_ inhibition primarily through a voltage-independent mechanism that cannot be significantly relieved by pre-pulses up to +120 mV [135,141,142]. This voltage-independent pathway is thought to be mediated by pertussis-sensitive Gα_i/o_ activity and requires the activity of c-Src kinase because inhibition of either leads to loss of Vc1.1-mediated Ca_V_ inhibition in both HEK293 cells and DRG neurons [135,142]. Interestingly, also unlike baclofen, Vc1.1 and RgIA were reported to have no effect on the activity of GIRK channels [143]. The α-conotoxin Vc1.1 also exhibits differential inhibition of Ca_V_ subtypes Ca_V_2.1 and Ca_V_2.3 heterologously expressed in HEK293 cells, with a preference for Ca_V_2.3 and little to no inhibition of Ca_V_2.1, compared with conventional agonists baclofen and GABA which inhibit both subtypes to a similar extent [141]. This study also provided further support to the role of c-Src-mediated inhibition of Ca_V_ which is thought to occur by phosphorylation of residues Y1761 and Y1765 in the α_1_ subunit, because mutation of these residues leads to loss of sensitivity to cVc1.1 [141]. Mutagenesis of GABA_B_R performed by Huynh et al. revealed alternate mechanisms by which this receptor triggers signal transduction in response to α-conotoxins versus baclofen. The R576D mutation in the GABA_B_R2 subunit, which inhibits G-protein activation by baclofen, does not affect Vc1.1 activity, suggesting a novel mechanism for stimulating Gα_i/o_ [142]. Conversely, truncation of the proximal C-terminus (PCT) of GBR1 leads to loss of sensitivity to Vc1.1 but does not affect baclofen signalling [142]. 

**Figure 1:**
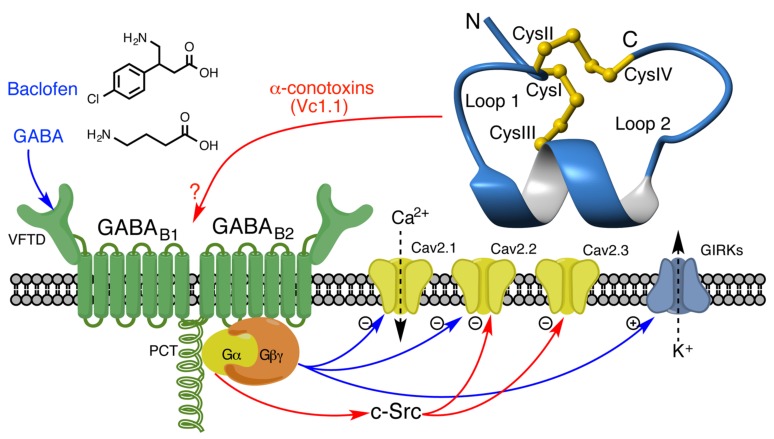
Modulation of ion channels by GABA_B_R agonists (blue) and α-conotoxins (red). Conventional GABA_B_R agonists such as baclofen and GABA bind to an orthosteric site on the extracellular Venus Fly Trap domain (VFTD) and are inhibited by the S246A and S270A mutations. Activated G_βγ_ subunit directly interacts with Ca_v_ channels and inhibits their activity by hyperpolarising activation threshold (voltage-dependent), with a preference for Ca_v_2.1 and Ca_v_2.2 subtypes. G_βγ_ activation in response to baclofen/GABA also activates G-protein coupled inwardly rectifying K^+^ channels (GIRKs). In contrast, inhibition of Ca_v_ channels by α-conotoxins such as Vc1.1 (pictured) is unaffected by S246A/S270A and these peptides do not compete with orthosteric ligands, suggesting an allosteric, but currently unknown, binding site. Furthermore, α-conotoxins primarily inhibit Ca_v_2.2 and Ca_v_2.3 channels through a novel mechanism involving the proximal C-terminus (PCT) domain and activation of the Gα_i/o_ subunit. c-Src kinase plays an important role in subsequent signal transduction which culminates in a voltage-independent inhibition of Ca_v_2.2 and Ca_v_2.3 by phosphorylation of tyrosine residues in the α1 subunit. α-Conotoxins also have no effect on GIRK activity.

Collectively, these studies indicate a proposed mechanism for α-conotoxin inhibition of Ca_V_s via GABA_B_R, which is outlined in Figure 1. The precise nature of α-conotoxin binding to GABA_B_R is currently unknown, but several lines of evidence suggest they interact allosterically to the VFTD binding site of classical agonists. Neither Vc1.1 nor RgIA displace the radiolabelled antagonist [^3^H]-CGP54626 in HEK293 cells [143] or DRG neurons [144], nor do they displace fluorescently labelled CGP54626-Red [145]. Furthermore, S246A and S270A mutations in the VFTD of GBR1, which inhibit agonist binding, do not affect the ability of Vc1.1 to inhibit Ca_V_s [142]. Molecular docking using the GABA_B_R crystal structure predicted important interactions between α-conotoxins and residues located in the ectodomain interface of the heterodimer [144], however, no experimental evidence exists for such an interaction between α-conotoxins and GABA_B_Rs. It is also noteworthy that Wright et al. failed to replicate a significant inhibitory effect on Ca_V_ currents in rodent DRG neurons following application of either Vc1.1 or RgIA [146], and Christensen et al. did not observe G-protein dissociation using a BRET assay, following application of a variety of α-conotoxins including Vc1.1 and AuIB to HEK293 cells heterologously expressing GABA_B_Rs [145]. These observations are inconsistent with the mechanism described above involving G-protein activation and therefore further investigation is required to reconcile these disparities and more accurately characterise the nature of GABA_B_R-dependent Ca_V_ inhibition by α-conotoxins. 

Although little is known regarding the structural and functional properties of *Conus* peptides that target the GABA_B_R, several important structure–activity relationships have been identified using analogues of α-conotoxins. The highly conserved loop 1 region (i.e., residues between CysII and CysIII) of α-conotoxins has been repeatedly identified as a key determinant of binding to nAChR subtypes [125,126], and these residues are also closely conserved between several GABA_B_R-targeting conotoxins, in particular Vc1.1 and RgIA, which share identical residues 1 to 8. The conserved proline in this loop is particularly important for GABA_B_R activity because replacement with hydroxyproline as in [P6O]Vc1.1 leads to loss of Ca_V_ inhibition in both DRG neurons [135] and colonic nociceptors [102]. Analogues of both Vc1.1 and RgIA in which the CysI–CysIII disulfide bond was replaced with a C=C (dicarba) bridge showed differential selectivity for Ca_V_ inhibition versus nAChRs, with the [II–IV]dicarba analogues favouring α9α10 inhibition and the [I–III]dicarba analogues favouring Cav2.2 inhibition [147,148]. Similarly, stabilising the hydrophobic core of cVc1.1 by replacing Cys2 and Cys8 with hydrophobic His and Phe residues does not perturb activity at the GABA_B_R [149]. Recently, Carstens et al. also performed structure–activity studies on conotoxins which inhibit Ca_V_ currents in DRG neurons using truncated analogues consisting of only the N-terminal tail and loop 1 residues of either Vc1.1/RgIA (G**C**SSDPR**C**) or Pu1.2/AuIB (GG**C**SSYPP**C**) braced by the CysI–III disulfide bond, denoted [Ser^3^]Vc1.1(1–8) and [Ser^4^]Pu1.2(1–9), respectively. Both analogues retained wild-type levels of Ca_V_ inhibition in rodent DRG neurons at 1 µM, revealing that loop 1 residues alone are sufficient for activation of GABA_B_R-dependent Ca_V_ inhibition. These loop 1 analogues also inhibited α7 nAChR currents in *Xenopus* oocytes, but lost activity at the α9α10 nAChR subtype. Interestingly, [Ser^3^]Vc1.1(1–8) retained analgesic properties in an in-vivo model of visceral mechanical hyperalgesia and was able to significantly inhibit the visceromotor response following colorectal distension when administered intracolonically (10 pmol) compared to vehicle [150], consistent with a GABA_B_R-dependent mechanism by which the parent peptide Vc1.1 inhibits colonic nociceptor firing [102]. 

The discovery of this alternate mechanism for reducing neuronal excitability in DRG neurons has led to some difficulty defining the relative contributions of nAChR and Ca_V_ inhibition toward the analgesic properties of α-conotoxins [143,144,151,152,153,154]. A major role for GABA_B_R/Ca_V_-dependent analgesia is supported by observations that natively expressed Vc1.1 containing two PTMs (hydroxyproline and γ-carboxyglutamic acid at positions 6 and 14, respectively), denoted vc1a, which is inactive at α3-, α4- and α7-containing nAChR subtypes [138,155] as well as the GABA_B_R [135] but equipotent at the α9α10 subtype compared to synthetic Vc1.1, does not reduce mechanical allodynia in a rat PNL model of neuropathic pain [151]. Similarly, the [P6O]Vc1.1 analogue which also does not inhibit Ca_V_ currents in DRG neurons has no effect on mechanical allodynia [151] and is unable to inhibit firing of colonic nociceptors [102]. Additionally, another GABA_B_R-targeting α-conotoxin, AuIB, which is selective for α3β4 and inactive at the α9α10 subtype, has a significant analgesic effect in rodent pain models [129,130]. Furthermore, blocking GABA_B_R activity by pretreatment with selective antagonists CGP 55845 or SCH 50911 abolishes the analgesic effects of Vc1.1 and AuIB in CCI, PNL and CVH models of pain [102,128,129]. Taken together, these studies implicate Ca_V_ inhibition via GABA_B_R as a key mechanism for reducing hyperalgesia and allodynia in a variety of neuropathic pain models. Interestingly, when Vc1.1, MII or AuIB were applied intrathecally, they did not inhibit EPSCs in dorsal horn neurons [130], unlike baclofen which evokes its physiological effects primarily through inhibition of these central neurons. However, since α-conotoxins exhibit analgesic properties when administered in peripheral tissues and conotoxins generally have little or no blood–brain barrier penetration, central activation of GABA_B_R may not be required for analgesia. A peripheral mode of action is consistent with the observations that α-conotoxins Vc1.1 and RgIA do not produce cognitive side effects or tolerance [127,129,130] and that peripherally acting GABA_B_R PAMs display analgesic activity in acute and chronic pain models but have no effect in cognitive behavioural tests [156,157]. This selectivity for inhibition of peripheral sensory neurons might bestow α-conotoxins with the advantage of greater therapeutic windows compared to conventional analgesics which are heavily limited by invasive injections, severe side effects, tolerance and addiction. Also, given the widespread co-expression of GABA_B_R and Ca_V_ subtypes throughout peripheral DRG neurons, particularly in the gut, and that dysregulation of GABAergic inhibition is a hallmark of pathological nociception, use of conotoxins to target this pathway is an attractive avenue for treating various neuropathies. 

In contrast, other evidence supports a less-substantial analgesic role of Ca_V_ inhibition compared to nAChR inhibition, in particular the α9α10 subtype which is the most selective target of RgIA and Vc1.1. Recently a synthetic analogue of RgIA, RgIA4, was developed as a selective α9α10 antagonist which showed no activity at other nAChR subtypes or GABA_B_R in vitro [133]. RgIA4 produced prolonged analgesia (up to three weeks) in a rat model of oxaliplatin-induced neuropathic pain as shown by reduction in mechanical hyperalgesia and cold allodynia [133,145]. Knockout of the α9 subunit mimicked the effects of RgIA4 treatment, suggesting the α9α10 nAChR is a primary target mediating the analgesic and protective effects, and that GABA_B_R activation is not required. In an earlier study using α9-knockout mice, the development of cold and mechanical allodynia occurred to a similar degree as wild-type, but development of chronic mechanical hyperalgesia was diminished [158]. Selective small-molecule α9α10 antagonists have shown reversal of chronic pain symptoms in CCI and chemotherapy-induced rodent models of neuropathic pain, supporting an analgesic role of α9α10 blockade [159,160]. Inhibition of α9α10 in immune cells has also been proposed as a mechanism by which α-conotoxins exert prolonged neuroprotective effects and contribute to functional recovery of injured nerves [122]. Overall, although a precise and undisputed analgesic pathway remains elusive, the current evidence suggests that both nAChR- and GABA_B_R-mediated mechanisms are likely to be involved in mediating the analgesic and neuroprotective effects of α-conotoxins, with relative contributions possibly dependent on the neuropathic pain model assessed. Despite the gaps in understanding of molecular mechanisms underlying α-conotoxins, they continue to be a promising class of lead molecules for the development of novel neuropathic pain therapeutics.

#### 3.2.2. Neurotensin Receptors Targeted by Contulakin-G

Although the majority of research and development of *Conus* peptides has focused on the conotoxin families, there also exist a number of disulfide-poor conopeptides which target GPCRs. One such peptide, contulakin-G, isolated from the venom of *Conus geographus*, contains 16 amino acids with two post-translational modifications, namely an N-terminal pyroglutamic acid (Z) and an *O*-glycosylated threonine at position 10 [161]. Contulakin-G (ZSEEGGSNTKKPYIL) shares high sequence homology with human neurotensin (ZLYENKPRRPYIL), particularly in the last six C-terminal residues, which form the pharmacophore of these peptides. In fact, NTS(8–13) alone is sufficient for receptor activation and the crystal structure of rat NTSR1 has been solved in complex with this peptide fragment [162]. Neurotensin is a neuromodulatory hormone that exerts its physiological effects through activation of the neurotensin GPCR subtypes, NTSR1 and NTSR2, as well as the single TM domain subtype, NTSR3 (also called SORT1). NTSRs are distributed throughout the mammalian CNS and modulation by neurotensin regulates a variety of neurological and hormonal processes including sensory and motor functions, temperature, hormone secretion, gut motility and neurological disorders [163,164]. Importantly, NTSRs also play a role in regulating nociception [165], and a number of studies have identified NTSR activation as a mechanism for inhibiting nociception in vivo [166,167,168]. 

Initial binding studies performed by Craig et al. demonstrated that the non-glycosylated [Thr^10^]-contulakin-G had 16-fold lower affinity for human NTSR1 compared to neurotensin, whereas the affinity of the native glycosylated form was nearly 686-fold lower than neurotensin, with similar trends observed for NTSR2 [161]. Contulakin-G was biologically active following intracerebroventricular injection at doses as low as 30 pmol (indicated by loss of motor function) comparable to 1000 pmol of neurotensin, whereas 300 pmol of [Thr^10^]-contulakin-G had no effect, suggesting that *O*-glycosylation increases potency in vivo, despite reducing affinity for both NTSR1 and NTSR2 in vitro [161]. Contulakin-G was later found to produce dose-dependent anti-nociceptive effects following intrathecal administration in both rodent (formalin test) and canine (thermal skin twitching) models of pain without significant loss of motor functions [169,170].

Recently, Lee et al. investigated the role of glycosylation in desensitisation of NTSR1 by NTS, contulakin-G and several synthetic analogues. The half-maximal desensitisation concentration (DC_50_) value for contulakin-G was 120 times greater than neurotensin (444 nM and 3.7 nM, respectively), whereas the DC_50_ of non-glycosylated [Thr^10^]-contulakin-G was 3-fold less than native contulakin-G (144 nM). Homology modelling using the rat NTSR1 crystal structure predicted loss of favourable interactions made by contulakin-G compared to [Thr^10^]-contulakin-G [171] consistent with a role of glycosylation in reducing activation and affinity for NTSRs [161]. One particular analogue, memantine–contulakin-G, in which glycosylated Thr10 was replaced with a Glu10-coupled memantine moiety, resulted in the least NTSR1 desensitisation (DC_50_ 1506 nM) but retained similar potency to the native peptide. Memantine–contulakin-G displayed anti-nociceptive properties in a mouse model of acute pain following systemic administration (intraperitoneal bolus) of 4 mg/kg as demonstrated by significantly decreased tail-flick latency [171]. Since one of the major caveats of extensive GPCR activation is the potential for desensitisation which can lead to reduced efficacy over time, analogues of contulakin-G with increased DC_50_ but similar potency might offer better therapeutic leads for treatment of chronic pain. Contulakin-G entered clinical trials under the name CGX-1160 for treatment of spinal cord injury pain. In phase IA trials, intrathecal infusion of CGX-1160 produced meaningful analgesia based on Gracely pain score (up to 63% reduction; EC_50_ 58.7 µg/mL) and was well tolerated at doses up 1000 µg/mL [172], showing promise as an analgesic therapeutic. 

#### 3.2.3. κ-Opioid Receptor Targeted by Conorphins

The endogenous opioid system regulates a variety of neurological processes in mammals and is a well-established analgesic pathway [44,45]. Of the three major OR subtypes (µ, κ and δ), most physiological effects are attributed to µOR modulation which is the major target of first-line analgesic opioid drugs such as morphine. However, µOR activation by opioids is also associated with severe side effects as well as the potential for addiction. The discovery and characterisation of the alternate κOR and δOR subtypes have revealed distinct pharmacological and physiological properties and offer alternate analgesic targets [44,173]. Notably, the κOR, a Gα_i/o_-coupled receptor which mediates the endogenous effects of the neuropeptide dynorphin A, has been implicated as therapeutic target for several disorders including pain [174], anxiety and addiction [175]. Activation of κOR mediates analgesia in numerous models of inflammatory [176,177,178,179] and neuropathic pain [180,181,182] with particular effectiveness in visceral pain [183]. Unlike µORs, analgesia induced by κOR agonists is not associated with systemic dose-limiting side effects of respiratory depression or constipation, but does have centrally mediated side effects such as dysphoria and sedation. Therefore, selectively targeting these receptors in peripheral tissues has attracted substantial interest as a mechanism for pain therapy.

Conorphin-T is a nine-amino-acid peptide (NCCRRQICC) that was recently identified by screening of the *Conus textile* venom duct cDNA library and displayed low micromolar affinity for the κOR and selectivity over other OR subtypes [184]. Using this peptide as a lead, Brust et al. synthesised and characterised an extensive array of rationally designed analogues to investigate the conorphin pharmacophore and derive highly selective and biologically stable κOR agonists as analgesic compounds. Comparison of affinity among analogues revealed a model for the conorphin pharmacophore which consists of an N-terminal aromatic residue favourably positioned by an adjacent proline, followed by a bis-arginine motif, a replaceable spacer residue, hydrophobic residue and an amidated C-terminal vicinal disulfide moiety. Analogue 39 (conorphin-1; Bz-PRRQ[CHA]CC-NH_2_) was the most potent conorphin derivative with 30,000-fold greater affinity for κOR and 16-times greater biological half-life in human serum than conorphin-T. Conorphin-1 was subsequently tested in a mouse model of CVH and while no effect was observed in healthy mice, this peptide produced a significant dose-dependent reduction in mechanosensory response of colonic afferents from CVH mice [184]. This effect was totally reversed in the presence of selective κOR antagonist norbinaltorphimine, consistent with the known role of κOR-mediated visceral analgesia [183] and its increased expression and activity in the CVH mouse model [185]. However, another study exploring the analgesic properties of conorphin-1 found that intraplantar administration (up to 200 µM) did not display significant analgesic activity in rodent models of acute (formalin-induced), inflammatory (Freund’s Complete Adjuvant- or carrageenan-induced) or neuropathic (cisplatin-induced) pain [186], suggesting that peripheral κOR activation may be a less viable approach for treating non-visceral types of pain. 

### 3.3. Other GPCRs Targeted by Venom Peptides with Potential Analgesic Properties

The peptides outlined above have demonstrated pain relieving properties in vivo in animal models of neuropathic pain, however, a range of other venom peptides target GPCRs which are not generally thought of as primary analgesic targets, but may play minor or secondary roles in moderating sensation of pain. Table 3 provides a summary of these receptor families and evidence for their role in producing analgesia. Although these venom peptides have not shown direct analgesic efficacy in vivo, their interactions with physiologically relevant central and/or peripheral pain targets leaves open the possibility of developing novel peptide ligands that act through these receptors. However, due to the other important endogenous functions of those GPCR families described in Table 3, thorough investigation and optimisation of pharmacology is required for their development as selective pain therapeutics. 

## 4. Summary and Conclusions

The widespread incidence of chronic pain and lack of effective and selective pharmacological compounds to treat pain syndromes has prompted significant interest in the discovery of novel therapeutic strategies. Venom peptides are renowned for their potent physiological effects and favourable pharmaceutical properties and are an abundant source of potential therapeutic compounds for diverse clinical indications including pain. Given that GPCRs are widely expressed on peripheral and central sensory neurons that constitute pain circuitry where they modulate diverse aspects of canonical pain signalling, and that dysfunctional GPCR activity is heavily implicated in the pathogenesis of chronic pain, these receptors are appealing therapeutic targets for pain. Despite this, in contrast to ion-channel-targeting peptides, relatively few GPCR-targeting venom peptides have been exploited as analgesic lead molecules. However, several peptides isolated from venoms of *Conus* species which target GPCRs have demonstrated in-vivo efficacy in the treatment of pain, with limited side-effect profiles, including the α-conotoxins which target the GABA_B_Rs, contulakin-G which targets NTSRs and conorphins which target the κOR. In all cases, structure–activity based approaches have proved invaluable for optimising the biopharmaceutical properties such as potency, selectivity and stability. In addition, a number of peptide toxins are known to target GPCRs with pain-modulating properties and, although they have not demonstrated direct in-vivo analgesia, may offer leads for deriving compounds which selectively regulate pain via GPCRs. Although several are currently in preclinical and clinical development, to date, no venom peptides that target GPCRs have been approved for treatment of pain. Several issues limit the progress of venom peptides as pharmaceuticals, such as differential potency between species, receptor subtype selectivity, biological instability and limited routes of administration, which must be addressed to optimise the pharmaceutical potential of these compounds. Nevertheless, the continuous advances in techniques used to characterise GPCRs and venom peptides, and the ever-evolving concepts relating to GPCR signalling, are likely to expedite the peptide-based drug-discovery process and ideally lead to safer and more efficacious pharmacological therapies for chronic pain.

## Figures and Tables

**Table 1:** Approved venom peptide drugs.

Peptide	Species	Pharmacological Target	Indication	Year Approved
Captopril	Brazilian pit viper (*Bothrops jararaca*)	Angiotensin-converting enzyme inhibitor	Hypertension	1981
Eptifibatide	Southeastern pygmy rattlesnake (*Sistrurus miliarius barbouri*)	Platelet glycoprotein IIb/IIIa receptor inhibitor	Unstable angina	1998
MVIIA (Ziconotide)	Cone snail (*Conus magus*)	Ca_V_2.2 inhibitor	Neuropathic pain	2004
Exenatide	Gila monster (*Heloderma suspectum*)	Insulin secretagogue	Type 2 diabetes mellitus	2005

**Table 2:** Pharmacology of representative α-conotoxins.

α-Conotoxin	Sequence ^a^	nAChR Selectivity	Cav2.2 IC_50_ (nM) ^b^	Analgesic Activity	References
Vc1.1	G**CC**SDPR**C**NYDHPEI**C**	α9α10 > α3β2 ~ α3β4	1.7	PNL, CCI, CVH	[122,129,138]
RgIA	G**CC**SDPR**C**RYR**C**R	α9α10 > α3β2 ~ α3β4	7.3	PNL, CCI, chemotherapy	[122]
Vc1a	G**CC**SDOR**C**NYDHPγI**C**	α9α10	Inactive	Inactive	[122,136]
ImI	G**CC**SDPR**C**AWR**C**	α7	Inactive	N.D.	[135,139]
PeIA	G**CC**SHPA**C**SVNHPEL**C**	α9α10 ~ α3β2	1.1	N.D.	[136]
AuIB	G**CC**SYPP**C**FATNPD**C**	α3β4	1.5	PNL, CCI	[129]
MII	G**CC**SNPV**C**HLEHSNL**C**	α3β2	Inactive	PNL	[129]

**N.**D., not determined; CCI, chronic constriction injury; PNL, partial nerve ligation; CVH, chronic visceral hypersensitivity. ^a^ amidated C-terminus; ^b^ tested in dorsal root ganglion neurons.

**Table 3:** Major G-protein coupled receptor (GPCR) families targeted by venom peptides.

GPCR Family	Venom Peptide Ligands	Subtype Selectivity (Gα Subunit)	Evidence for Analgesic Role	References
Muscarinic acetylcholine receptor	MT1 MT2 MT4 MT5 MT7	M1 (Gα_q_)	Expressed throughout peripheral nociceptive and central nerves and are dysregulated in pain conditions. Various subtype selective and non-selective mAChR agonists are analgesic in rodent models of pain.	[73,187,188,189,190,191,192]
	MT3 MT6	M4 (Gα_i_)		
	MTLP-1	M3 (Gα_q_)		
α-Adrenergic receptor	*ρ*-Da1a *ρ*-TIA	α_1A_ (Gα_q_) α_1B_ (Gα_q_)	Widely expressed on nociceptors and central pathways and mediate descending inhibition. Several α2-selective agonists exhibit analgesic properties in rodents	[193,194,195,196]
	*ρ*-Da1b MTα MT1	α_2A_ (Gα_i_) α_2A_ α_2B_ (Gα_i_)		
	MT3	α_1B_ ~ α_2A_		
Oxytocin/vasopressin receptor	Conopressin-G Conopressin-S Conopressin-T	OTR (Gα_q_) OTR V_1a_ (Gα_q_) > OTR	Oxytocin acting via OTR and V_1a_ is analgesic in animal models of pain.	[197,198,199]
Opioid receptor	BmK-YA	δOR (Gα_i_)	Involved in presynaptic control of nociceptive inputs onto dorsal horn. Administration of δ-opioid agonists produces anti-allodynia in chronic pain models.	[173,200,201,202]
Endothelin receptor	SRTX-a SRTX-b SRTX-c	ET_A_, ET_B_ (Gα_q_)	Distributed in central and peripheral pathways. Implicated as a regulator of acute and chronic pain.	[203,204]
	S6c	ET_B_ > ET_A_		
Neuropeptide FF receptor	CNF-Sr1 CNF-Sr2 Vc1	None (Gα_i_)	Important role in modulating pain signalling in the CNS.	[205,206,207]
